# The potential for Ghana to become a leader in the African hemp industry

**DOI:** 10.1186/s42238-023-00205-9

**Published:** 2023-10-30

**Authors:** Richard Quansah Amissah

**Affiliations:** Department of Biomedical Sciences, University of Guelph, 50 Stone Road East, Guelph, ON N1G 2W1 Canada

**Keywords:** Ghana, Hemp, African hemp industry, Cultivation, Sustainable development

## Abstract

**Background:**

Global interest in hemp cultivation and utilization is on the rise, presenting both challenges and opportunities for African countries. This article focuses on Ghana’s potential to establish a thriving hemp sector, considering its favorable climate, abundant agricultural resources, and existing policies and programs that support the growth and advancement of the agricultural sector, as well as agro-processing and value addition.

**Main body:**

Ghana’s recent decriminalization of cannabis with low tetrahydrocannabinol (THC) levels marks a progressive step, unlocking opportunities for research, commercial production, and innovation in hemp-related sectors. This regulatory change paves the way for the development of textiles, construction materials, and wellness products derived from hemp. However, the African hemp industry faces various simultaneous challenges, including pest management, absence of regulatory frameworks, limited research, inadequate infrastructure, and lack of access to finance and investment capital for small-scale farmers. Fortunately, several countries that have legalized hemp cultivation and processing have found innovative solutions to these challenges through the use of integrated pest management strategies, establishing collaborations with international standards organizations, implementing public–private partnerships, offering tax incentives for investors, and providing low-interest loans and credit facilities for small-scale farmers. Ghana can draw inspiration from these successful approaches and adapt them to its own context to foster the growth of the hemp industry.

**Conclusion:**

By capitalizing on its strengths and addressing the challenges it is likely to face while developing its hemp industry, Ghana can position itself as a leader in the African hemp industry. This position of leadership would not only drive economic growth, but also create job opportunities and foster sustainable development through responsible hemp cultivation and utilization.

## Introduction

Industrial hemp is a variety of the Cannabis sativa plant species that is specifically grown for its industrial uses and derived products. Unlike marijuana, which is also a variety of Cannabis sativa, industrial hemp has a much lower concentration of the psychoactive compound tetrahydrocannabinol (THC), usually less than 0.3% (Adhikary et al. 2021). It is a versatile and renewable resource with a wide range of applications such as the production of fiber (Shahzad 2012), building materials (Collet and Pretot 2014), biofuel (Prade et al. 2011), food and nutrition (Callaway 2004), body care products (Leson et al. 2001), and animal bedding (Hillig 2004). Hemp also offers numerous environmental benefits including phytoremediation (Golia et al. 2023), renewable energy production (Struik et al. 2000), carbon sequestration (Beerling et al. 2018), reduced water and pesticide use (Callaway 2004; Fortenbery and Bennett 2004), and improved soil fertility (Kraenzel et al. 1998; Amaducci et al. 2015). Due to its numerous applications and environmental benefits, industrial hemp has gained significant interest in recent years, leading to increased cultivation and research worldwide.

The global hemp industry has experienced significant growth in recent years, driven by increasing demand for hemp-derived products and the legalization of hemp cultivation in various countries. The global hemp market was valued at approximately USD 4.6 billion in 2019 and is expected to reach USD 13.03 billion by 2026, growing at a compound annual growth rate of between 13.7% and 16.7% during 2019–2025 (Vivek 2022). The production of hemp has expanded rapidly, with China, Canada, and the European Union being the largest producers (Cherney and Small 2016). The USA has also seen a surge in hemp cultivation since the passage of the 2018 Farm Bill, which legalized hemp production at the federal level (Johnson et al. 2018). The hemp industry’s growth can be attributed to several factors, including increasing consumer awareness of the health benefits of hemp-derived products, such as cannabidiol (CBD) oil, and the growing demand for sustainable and eco-friendly materials (Fortenbery and Bennett 2004). Additionally, technological advancements in hemp processing have led to the development of new applications, further driving market growth (Kraenzel et al. 1998). Hemp-derived CBD has emerged as a significant market segment, with applications in pharmaceuticals, nutraceuticals, and personal care products (Pavlovic et al. 2018). Hemp fibers have also gained traction in the textile industry due to their durability, sustainability, and biodegradability (Keller 2003). The automotive industry has shown interest in using hemp-based materials for lightweight and eco-friendly components (Pickering et al. 2016). Overall, the global industrial hemp industry is expected to continue growing in the coming years, driven by increasing demand for hemp-based products and favorable regulatory environments in many countries.

The objectives of this paper are to highlight the potential of industrial hemp in enhancing Ghana’s economy, identify the current challenges faced by the African hemp industry, and propose strategies for Ghana, a West African country, to overcome these obstacles and become a leader in the African hemp industry. While this report primarily focuses on Ghana, the lessons and suggestions presented are applicable to other countries within and outside Africa.

### Historical background of hemp production in Africa

In Africa, the history of hemp production can be traced back to ancient Egypt, where it was used for various purposes, including the production of ropes, textiles, and sails for boats, while the seeds were consumed as food and the oil was used for medicinal purposes (Clarke and Merlin 2013). It was introduced into other African countries through trade with Arabs (Toit 1976). The expansion of European colonial powers in the nineteenth and twentieth centuries further contributed to the spread of hemp across the continent, as they introduced new cultivars and cultivation techniques (Clarke and Merlin 2013). In the post-colonial period, hemp production in Africa faced several challenges, including competition from synthetic fibers, restrictive drug policies, and a lack of investment in research and development (Carus and Sarmento 2016). As a result, hemp production declined in many African countries, and the cultivation of indigenous African hemp varieties was largely abandoned (Clarke and Merlin 2013). However, in recent years, there has been a resurgence of interest in hemp production in Africa.

### Overview of the current state of the African hemp industry

Several African countries are actively involved in hemp production, including South Africa, Lesotho, Malawi, and Zimbabwe (Duvall 2019). The favorable climate and fertile soils in these tropical regions provide ideal conditions for hemp cultivation (Wimalasiri et al. 2021), resulting in high yields and quality fiber.

In South Africa, hemp cultivation is subject to various laws and regulations, which have evolved over time. In 1992, the Drugs and Drug Trafficking Act banned all forms of cannabis cultivation, including hemp (South African Government 1992). However, the Medicines and Related Substances Act was amended in 2017 to allow for the cultivation of cannabis for medicinal purposes under strict licensing conditions, and in September 2018, the Constitutional Court decriminalized the private use, possession, and cultivation of cannabis for personal consumption, but this did not directly impact hemp cultivation for industrial purposes (Constitutional Court of South Africa 2018). The 2019 guidelines released by the Department of Agriculture, Forestry, and Fisheries marked a significant step towards the legalization of hemp cultivation in South Africa, with a THC limit of 0.2% (Grower IQ 2023). On the 29th of October, 2021, the Minister of Agriculture, Land Reform, and Rural Development announced the opening of application processes for hemp permits, which would allow the importation, exportation, and cultivation of hemp (South African Government 2021).

In 2017, Lesotho became the first country in Africa to grant licenses for the cultivation of marijuana for medicinal and scientific purposes (Forbes Africa 2022; Uwakonye 2020). The country’s government approved the production of medical marijuana to create jobs and boost the economy, and several companies have been licensed to cultivate and process marijuana. Lesotho’s favorable climate and high altitude make it an ideal location for growing high-quality cannabis, and the country has since become a major hub of industry development for cannabis and industrial hemp in Africa (Powell 2020).

Hemp cultivation in Malawi has experienced significant changes in recent years, with the government recognizing its potential for economic growth and environmental sustainability. In 2020, Malawi passed the Cannabis Regulation Act, which legalized the cultivation, processing, and sale of industrial hemp and medicinal cannabis (Global Development 2020; Bandawe 2022). Prior to this, cannabis cultivation was illegal under the Noxious Weeds Act of 1936 and the Dangerous Drugs Act of 1956 (Bandawe 2022). The 2020 legislation was implemented to diversify the country’s agricultural sector, create employment opportunities, and boost foreign exchange earnings. The Act established the Cannabis Regulatory Authority, responsible for issuing licenses and regulating the industry (Global Development 2020). Industrial hemp cultivation in Malawi is subject to strict regulations, including a maximum THC content of 0.3% and mandatory registration of farmers. Additionally, the government has implemented measures to ensure that hemp cultivation does not negatively impact the environment or local communities (Global Development 2020).

In recent years, Zimbabwe has made significant strides in re-establishing its hemp industry, with the government legalizing the cultivation of industrial hemp in 2019, which could lead to increased research and investment in the sector, focusing on the development of improved hemp varieties, agronomic practices, and value-added products (Hemp Today 2023). The current state of the Zimbabwean hemp industry is characterized by a growing interest from local and international stakeholders, as well as the establishment of pilot projects and commercial farms aimed at boosting the country’s agricultural output and economic growth (Hemp Today 2023).

### Challenges facing the African hemp industry

One of the primary challenges facing the hemp industry in Africa is the legal restrictions imposed by various governments. Despite the global trend towards the legalization of industrial hemp, many African countries still maintain strict regulations on its cultivation, evidenced by the fact that only a handful of African countries have legalized hemp cultivation (Duvall 2019). This has limited the expansion of the industry and stifled research efforts aimed at understanding the crop’s potential in the region (Carus and Sarmento 2016). Another significant challenge is the lack of infrastructure and investment in the hemp industry. Many African countries lack the necessary processing facilities and equipment (Fortune Business Insights 2021). This has limited the potential for the industry to create jobs and generate income for local communities. There is also a lack of transportation networks and storage facilities, which hinders the ability of farmers to efficiently transport their crops and process them into value-added products (Fox and Signe 2022). Additionally, the African hemp industry faces limited access to markets due to the lack of established supply chains and export infrastructure (Lowitt 2020), especially since only 12 African countries allow the growing of industrial hemp. This limits the ability of farmers and entrepreneurs to sell their products in global markets, where demand for hemp products is high. The lack of clear regulations and policies on hemp cultivation and processing in some African countries makes it difficult for farmers and investors to engage in the industry (UNCTAD 2022). Overcoming these challenges will require concerted efforts from governments, researchers, and industry stakeholders to create a conducive environment for the growth and development of the hemp industry in the region.

### Hemp industry potential in Ghana

#### Overview of Ghana’s agricultural sector

Ghana’s agricultural sector plays a crucial role in the country’s economy and food security. The contribution of agriculture to the country’s gross domestic product (GDP) has ranged from 23.6% in 2014 to 20.5% in 2017 (Owusu et al. 2021). The main crops grown in Ghana include cocoa, oil palm, rubber, cotton, and various food crops such as maize, rice, yam, cassava, and plantain (Ministry of Food and Agriculture 2019). Cocoa is the most important cash crop, contributing to about 30% of the country’s total export earnings (Roessler 2022; Wessel and Quist-Wessel 2015). Ghana is the second-largest cocoa producer globally, with the crop mainly grown in the Ashanti, Western, Central, and Eastern regions (Bryant and Mitchell 2021). Smallholder farmers dominate Ghana’s agricultural sector, with about 90% of farms being less than 2 ha in size (Food and Agriculture Organization 2018). Livestock production is another important component of Ghana’s agricultural sector, mainly comprising poultry, cattle, sheep, goats, and pigs (Ministry of Food and Agriculture 2019). Fisheries also play a vital role in Ghana’s agricultural sector, providing livelihoods for over 2 million people and contributing to about 5% of the country’s GDP (Tall and Failler 2012). The fisheries subsector is divided into marine and inland fisheries, with marine fisheries accounting for about 80% of the total fish production (Nunoo et al. 2014). However, overfishing, illegal fishing practices, and climate change have led to a decline in fish stocks, threatening the sustainability of the subsector (Asiedu et al. 2019). The government of Ghana has implemented various policies and programs to promote the growth and development of the agricultural sector. These include the Planting for Food and Jobs (PFJ) program, which aims to increase food production and create employment opportunities through the provision of subsidized inputs and extension services (Ministry of Food and Agriculture 2017). Additionally, the One District, One Factory (1D1F) initiative seeks to promote agro-processing and value addition, thereby reducing post-harvest losses and increasing farmers’ incomes (Ghana Investment Promotion Centre 2020).

#### Hemp cultivation potential in Ghana

Ghana has a significant potential for hemp cultivation due to its favorable climate, fertile soil, and a growing interest in the economic benefits of the crop. The country’s tropical climate, characterized by a bimodal rainfall pattern and average temperatures ranging between 21 and 32 °C, provides an ideal environment for hemp growth (Rhebergen et al. 2016). Ghana’s agricultural sector could benefit from the introduction of hemp as a cash crop. The cultivation of hemp in Ghana could be facilitated by the country’s abundant cultivable land, which accounts for approximately 42% of the total land area (Frenken 2005). The Volta, Ashanti, and Brong-Ahafo (which has been now split into the Bono, Ahafo, and Bono East) regions, in particular, have been identified as suitable areas for hemp cultivation due to their fertile soils and adequate rainfall (Quansah Amissah 2022). Additionally, hemp is a low-input crop, requiring minimal amounts of water, fertilizers, and pesticides, making it an environmentally friendly and sustainable option for Ghanaian farmers (Carus and Sarmento 2016). The potential for hemp cultivation in Ghana is further supported by the country’s existing agricultural infrastructure and skilled labor force. However, there are some challenges to the widespread adoption of hemp cultivation in Ghana. One of the primary concerns is that even though it has been decriminalized, its use and cultivation remain illegal unless one has the necessary license to do so, and none has been issued to date (Owusu et al. 2021; Quansah Amissah 2022).

The first report of illegal cannabis cultivation in Ghana dates back to 1960. Between that year and 1980, this illicit activity experienced significant growth (Bernstein 1999). Most of the cannabis grown in the country was cultivated in the Eastern, Ashanti, Bono, Ahafo, and Bono East regions, where farmers, recognizing its profitability, planted it alongside other crops such as cassava, tomatoes, okra, and cocoa. In fact, Ghana is renowned for producing high-quality cannabis due to its high THC content, and a majority of this premium cannabis is exported, establishing the country as a leading cannabis exporter within the region of West Africa (Bernstein 1999). As a result, Ghana has a lengthy history of cannabis cultivation, which can be particularly valuable, especially in light of the recent legalization of hemp cultivation in the country. This history can aid in identifying which regions in the country are conducive to hemp cultivation and which strains will thrive in these areas. Furthermore, the indigenous knowledge and experience gained during this period, as well as techniques developed for cannabis cultivation can be adapted for hemp cultivation to optimize yields and revenue for farmers and all parties involved.

#### Legal framework for hemp cultivation and trade in Ghana

The legal framework for hemp cultivation in Ghana has undergone significant changes in recent years, reflecting the growing global interest in the crop’s industrial and medicinal applications. Historically, hemp has been classified as an illegal narcotic in Ghana due to its association with marijuana and the psychoactive compound THC (Quansah Amissah 2022). However, recent legislative efforts have sought to differentiate between industrial hemp, which has low THC levels, and marijuana, which has higher THC concentrations (Adhikary et al. 2021). In March 2020, the Parliament of Ghana passed the Narcotics Control Commission Act, 2020 (Act 1019), which established the Narcotics Control Commission as the regulatory body responsible for overseeing the cultivation, production, and distribution of narcotic substances, including hemp (Hemp Industry Daily 2020), even though the Supreme court of Ghana recently struck out and described as unconstitutional Section 43 of Act 1019, which gave the Minister of Interior the power to grant licenses for hemp cultivation (Agyemang 2023). The Act also redefined hemp with a THC content of 0.3% or less as a non-narcotic substance, paving the way for the development of a legal framework for industrial hemp cultivation in the country (Owusu et al. 2021). Following the passage of Act 1019, the Ghanaian government has been working on drafting legislation to regulate the cultivation and use of industrial hemp. The proposed legislation aims to promote the economic potential of hemp while ensuring strict regulatory controls to prevent the illicit use of the crop. In addition to the national legal framework, Ghana is also subject to international conventions and agreements related to hemp and narcotic substances. As a signatory to the United Nations Single Convention on Narcotic Drugs (1961), Ghana is obligated to implement measures to control the cultivation, production, and distribution of narcotic substances, including hemp (Quansah Amissah 2022). The recent legislative efforts to regulate industrial hemp cultivation in Ghana are in line with the country’s international commitments.

#### Current and potential market opportunities for Ghanaian hemp

One significant market opportunity for Ghanaian hemp lies in the textile industry. Hemp fibers are known for their strength, durability, and eco-friendliness, making them an attractive alternative to traditional textile materials like cotton (Gedik and Avinc 2020; Crini et al. 2020). Another promising area for Ghanaian hemp is the food and beverage sector. Hemp seeds are rich in essential fatty acids, proteins, and other nutrients, making them a valuable addition to various food products (Callaway 2004). The construction industry also presents potential market opportunities for Ghanaian hemp. Hempcrete, a sustainable building material made from hemp hurds and lime has excellent insulation properties and can help reduce energy consumption in buildings (Bevan and Woolley 2008). With the rapid urbanization and growing construction industry in Ghana (Ahmed et al. 2014), the demand for sustainable building materials like hempcrete is expected to increase. In the medicinal and wellness sector, Ghanaian hemp can capitalize on the growing interest in CBD products. CBD has been shown to have potential therapeutic effects for various conditions, including epilepsy, anxiety, and chronic pain (Blessing et al. 2015), and could therefore be investigated to identify alternative treatments for diseases (such as epilepsy and various types of cancers) that are prevalent in the country (Quansah Amissah 2022). There are several potential market opportunities for Ghanaian hemp, including textiles, food and beverages, construction, and medicinal products. As the global demand for hemp products continues to grow, Ghana is well-positioned to capitalize on these opportunities and establish itself as a significant player in the hemp industry.

#### Potential economic, social, and environmental benefits of hemp production in Ghana

Hemp production in Ghana has the potential to bring about significant economic, social, and environmental benefits. The cultivation of hemp in Ghana could create new job opportunities in agriculture, processing, and manufacturing sectors, thereby contributing to the country’s economic growth. Hemp production can also contribute to rural development and poverty alleviation in Ghana, as the newly created jobs could lead to increased income and improved living standards for rural communities (Fortenbery and Bennett 2004). Additionally, hemp can be grown on marginal lands, which means that it can be cultivated without competing with food crops for arable land (Salentijn et al. 2015). This could lead to increased agricultural productivity and income for Ghanaian farmers. By tapping into the global hemp market, Ghana could significantly boost its export revenues. Moreover, hemp is a low-input crop that requires minimal amounts of water, pesticides, and fertilizers (Amaducci et al. 2015). This makes it an affordable and accessible crop for smallholder farmers, who often face financial constraints and limited access to agricultural inputs. Hemp production can have positive environmental impacts in Ghana. Due to its deep root system, hemp can improve soil structure and fertility, thereby promoting sustainable land management practices (Tang et al. 2017).

### How Ghana can overcome the challenges facing the African hemp industry

#### Lessons from other countries’ experiences with hemp cultivation and trade

Firstly, the success of hemp cultivation in countries like China, Canada, and the USA can be attributed to the development of well-regulated and supportive legal frameworks (Cherney and Small 2016). Secondly, investment in research and development is crucial for the growth of the hemp industry. Countries like France and the Netherlands have made significant advancements in hemp breeding and agronomic practices through dedicated research programs (Amaducci et al. 2015). Thirdly, the establishment of strong value chains is essential for the success of the hemp industry. Countries like Germany and Italy have developed robust value chains by promoting the use of hemp in various industrial applications, such as textiles, construction materials, and bioplastics (Bocsa and Karus 1998). Fourthly, the importance of farmer education and extension services cannot be overstated. Countries like Australia and South Africa have successfully introduced hemp cultivation by providing farmers with the necessary knowledge and skills to grow the crop (Lisson et al. 2000; Van der Werf et al. 1994). Ghana can learn valuable lessons from other countries’ experiences with hemp cultivation and trade. By implementing these strategies, Ghana can unlock the full potential of the hemp industry and contribute to the country’s economic growth and sustainable development.

#### Overcoming challenges faced by other African countries involved in the hemp industry

As previously mentioned, the African hemp industry faces various challenges. In order for Ghana to emerge as a leader in this industry, it must develop innovative solutions to overcome these challenges. The following sections will discuss some of the challenges faced and propose possible ways for Ghana to address them.

1. Legal restrictions imposed by governments: To overcome legal restrictions, Ghana can follow the example of countries like South Africa and Lesotho that have legalized hemp cultivation for industrial and medicinal purposes (Owusu et al. 2021). The government can establish a regulatory framework that allows for the controlled cultivation of industrial hemp with low THC content (< 0.3%) and issue licenses to farmers and businesses involved in the industry. This will help create a legal and regulated market for hemp products in Ghana as well as address the concerns of policymakers and stakeholders. In Canada, the Industrial Hemp Regulations under the Cannabis Act provide a clear framework for the cultivation, processing, and sale of industrial hemp (Government of Canada 2018). The European Union has also established guidelines for industrial hemp cultivation (Farinon et al. 2020). In the USA, the 2018 Farm Bill legalized industrial hemp cultivation and established a regulatory framework under the United States Department of Agriculture (Johnson et al. 2018; United States Department of Agriculture 2019). These regulations could be used as templates in establishing regulatory frameworks in Ghana.
2. Lack of infrastructure and investment: Ghana can attract investment in the hemp industry by offering incentives such as tax breaks, low-interest loans, and grants to businesses involved in hemp cultivation, processing, and manufacturing. The government can also invest in infrastructure development, such as transportation and storage facilities, to support the growth of the industry. A good example is the Canadian government’s investment in the hemp industry, which has led to significant growth in recent years (Small 2016). Ghana could also establish public–private partnerships (PPPs) to develop the necessary infrastructure for industrial hemp cultivation, processing, and distribution. PPPs can help mobilize private sector resources and expertise to build the required infrastructure, such as processing facilities, storage, and transportation networks. By attracting investment, Ghana can stimulate the growth of the hemp industry and create jobs.
3. Limited research on hemp cultivation: To address this challenge, Ghana can establish research partnerships with international institutions and organizations, such as the European Industrial Hemp Association and the International Hemp Building Association (Karus 2004; Lawrence 2015). These partnerships can help facilitate knowledge exchange and capacity building in hemp cultivation and processing. Additionally, the government can allocate funding for research and development in the hemp industry, as seen in the United States with the 2018 Farm Bill (Johnson et al. 2018). The government of Ghana could establish dedicated research centers focused on industrial hemp cultivation. These centers can collaborate with local universities, international research institutions, and private companies to conduct extensive research on hemp varieties, cultivation techniques, and processing methods suitable for the African climate and soil conditions.
4. Climate change: To mitigate the effects of climate change, Ghana can promote the use of climate-resilient hemp varieties and sustainable farming practices, such as crop rotation, organic farming, and water conservation techniques (Padmavathy and Poyyamoli 2011) by implementing training programs and support services to help farmers adapt to climate change. The government can also allocate resources for research aimed at developing hemp varieties that are better adapted to changing climatic conditions. These varieties should be able to withstand extreme weather conditions, such as drought, flooding, and temperature fluctuations. The Australian government serves as an example in this regard (McPartland et al. 2000).
5. Pest management: Ghana can adopt integrated pest management (IPM) strategies to control pests and diseases in hemp cultivation since pest management is a significant challenge in Africa (Makundi 2006). IPM involves the use of biological, cultural, and chemical methods to manage pests and diseases in a sustainable manner (Kogan 1998). Examples of successful IPM implementation that focuses on crop rotation, field scouting, and the use of biocontrol agents can be found in countries like the USA and Canada (Bocsa and Karus 1998; McPartland et al. 2000). The European Union also promotes IPM as a key component of sustainable agriculture. France and Germany have adopted these strategies in hemp cultivation (Matyjaszczyk 2015).
6. Lack of clear regulations and policies on hemp cultivation and processing: Ghana can address this challenge by establishing a dedicated regulatory body responsible for overseeing the industry. This body can develop guidelines and standards for hemp cultivation, processing, and product quality. One existing organization in Ghana that may be capable of fulfilling this role is the Center for Plant Medicine Research (Quansah Amissah 2022). Examples from other countries can provide guidance. For instance, in Canada, the Industrial Hemp Regulations under the Cannabis Act offer a comprehensive framework for the cultivation, processing, and sale of industrial hemp (Government of Canada 2018). Similarly, in the USA, the 2018 Farm Bill established a regulatory framework under the United States Department of Agriculture (Johnson et al. 2018).
7. Lack of standardized testing protocols for hemp products: To address this challenge, Ghana can collaborate with international organizations, such as the International Organization for Standardization, to develop and adopt standardized testing methods for hemp products. Ghana could also establish a national hemp testing and certification body. This will ensure that all hemp products meet quality and safety standards before entering the local and international markets.
8. Stigma associated with cannabis and hemp: To overcome this, Ghana can initiate public awareness campaigns. These campaigns should aim to educate diverse stakeholders, including policymakers, farmers, and the general public, about the distinctions between industrial hemp and marijuana, as well as the economic, environmental, and health benefits of hemp. By doing so, public perception can be transformed, fostering a more favorable environment for the hemp industry. Canada serves as a successful example of implementing public awareness campaigns to educate citizens about the differences between hemp and marijuana (Small and Marcus 2002).
9. Lack of access to finance and investment capital for small-scale farmers and entrepreneurs: Ghana can establish financial support programs, such as low-interest loans, grants, and credit facilities, specifically targeted at small-scale farmers and entrepreneurs in the hemp industry. This can help them access the necessary capital to invest in hemp cultivation and processing. A good example is the United States’ Small Business Administration, which provides financial assistance to small businesses in various industries, including agriculture (Solomon and Carney 1985).

Addressing these challenges may not be a simple task and could demand a collaborative effort involving various local and international organizations. It may also necessitate substantial technical and financial resources to overcome them. Nonetheless, it is through effectively addressing these challenges that Ghana can solidify its position as a leader in the African hemp industry.

After discussing the potential benefits of Ghana’s hemp industry for the country and how addressing the aforementioned challenges could help establish Ghana as a leader in the African hemp industry, it is crucial to acknowledge that these benefits also bring potential risks, especially for the youth, who might gain easier access to illegally produced cannabis from farmers. Addressing this issue is essential to prevent undesired consequences following the legalization of hemp cultivation in the country. To deter farmers from taking advantage of the burgeoning hemp industry to produce illegal cannabis, the government should establish a licensing and registration system for hemp farmers. This system will ensure that only licensed farmers are permitted to cultivate hemp and that they are producing hemp with a maximum THC content of 0.3% (Owusu et al. 2021; Quansah Amissah 2022). Additionally, a monitoring and testing system should be established to ensure the hemp produced meets the required standards. However, these regulations will only be effective if they are rigorously enforced. Therefore, they should be enforced through partnerships with law enforcement agencies in the country. To mitigate the potential risk of cannabis use among the youth, policies and programs for substance abuse prevention need to be established. These initiatives should focus on educating the youth about the harmful effects of cannabis use and promoting healthy lifestyles (Tetteh et al. 2012). The harmful effects of substance use could also be integrated into the education curricula to raise awareness among students (Adu-Mireku 2003). Furthermore, effective regulatory frameworks to monitor the production and use of hemp products, as well as the monitoring of the industry to ensure compliance with regulations will be necessary (Quansah Amissah 2022). These measures can help mitigate the potential risk of farmers taking advantage of the growing hemp industry in the country to illegally produce cannabis and reduce the risk of cannabis abuse among the population, especially the youth.

## Conclusion

The African hemp industry is relatively young but faces numerous concurrent challenges. These challenges include the absence of regulatory frameworks, inadequate infrastructure, limited research, and insufficient capital for small-scale farmers. Consequently, the continent struggles to establish itself as a global leader in the hemp industry. While Ghana has recently decriminalized hemp cultivation, it still has a long way to go in supporting the continent’s quest for industrial hemp leadership. Ghana must develop innovative solutions to address the challenges faced not only by itself but also by other African countries. These solutions encompass establishing clear and comprehensive regulatory frameworks, investing in infrastructure through partnerships, collaborating with reputable international organizations to establish robust research centers, and providing financial incentives and training for small-scale farmers, who constitute the majority of farmers in the country. Only by implementing these measures can Ghana establish a thriving industrial hemp industry and assume a leadership position within the African hemp industry.

## Data Availability

Not applicable.

## Abbreviations

CBD: Cannabidiol
THC: Δ^9^-Tetrahydrocannabinol
GDP: Gross domestic product
PPPs: Public-private partnerships
IPM: Integrated pest management
